# Supplementary material for: Spatio-spectral 4D coherent ranging using a flutter-wavelength-swept laser
Source: Nat Commun. 2024 Feb 6;15:1110. doi: 10.1038/s41467-024-45297-w (PMC10847489; doi:10.1038/s41467-024-45297-w)
Supplement: Supplementary file 1 — Supplementary Information [file 41467_2024_45297_MOESM1_ESM.pdf]

Supplementary Information

Spatio-spectral 4D Coherent Ranging Using a Flutter-wavelength-swept Laser

Dawoon Jeong[1,2], Hansol Jang[3], Min Uk Jung[1,2], Taeho Jeong[4], Hyunsoo Kim[5], Sanghyeok Yang[5], Janghyun Lee[5], Chang-Seok Kim[1,2,*]

[1]Department of Cogno-Mechatronics Engineering, Pusan National University, Busan, 46241, Korea

[2]Engineering Research Center for Color-Modulated Extra-Sensory Perception Technology, Pusan National University, Busan, 46241, Korea

[3]Ground Technology Research Institute, Agency for Defense Development, Daejeon, 34186, Korea

[4]Energy Device Research Team, Hyundai Motor Company, Uiwang, Gyeonggi, 16082, Korea

[5]Electromagnetic Energy Materials Research Team, Hyundai Motor Company, Uiwang, Gyeonggi, 16082, Korea

*Corresponding Author: ckim@pusan.ac.kr (Chang-Seok Kim)

Supplementary Note 1. Spatio-spectral coherent LiDAR based on wavelength-swept laser

The main differences between spatio-spectral coherent light detection and ranging (LiDAR) based on a flutter-wavelength-swept laser (FWSL) and conventional wavelength-swept lasers are summarized in Supplementary Fig. 1. In contrast to conventional wavelength-swept lasers, which typically offer unidirectional wavelength sweeps over the entire wavelength bandwidth, the proposed FWSL enables independent bidirectional wavelength modulation during continuous wavelength sweep. Accordingly, the flutter-wavelength modulation segment containing both distance and velocity information was instantly and sequentially sampled, substantially extending the measurable distance. To compare the different schemes effectively, the laser source specifications and the resulting LiDAR system specifications were quantitatively calculated. We assumed a hypothetical case in which our FWSL operated as typical wavelength-swept lasers, as shown in Supplementary Fig. 1b. In this hypothetical case, we kept the major settings such as the wavelength sweep bandwidth, fast axis scan rate, electrical bandwidth, and imaging pixels (H × V) size, the same as in our actual system. Moreover, the spectral axis was assumed to be the fast axis, according to the previous studies[1,2]. As listed in Supplementary Table 1, the FWSL enables simultaneous distance and velocity measurements over relatively long distances with the same sweep bandwidth and electrical bandwidth, owing to its unique operation.

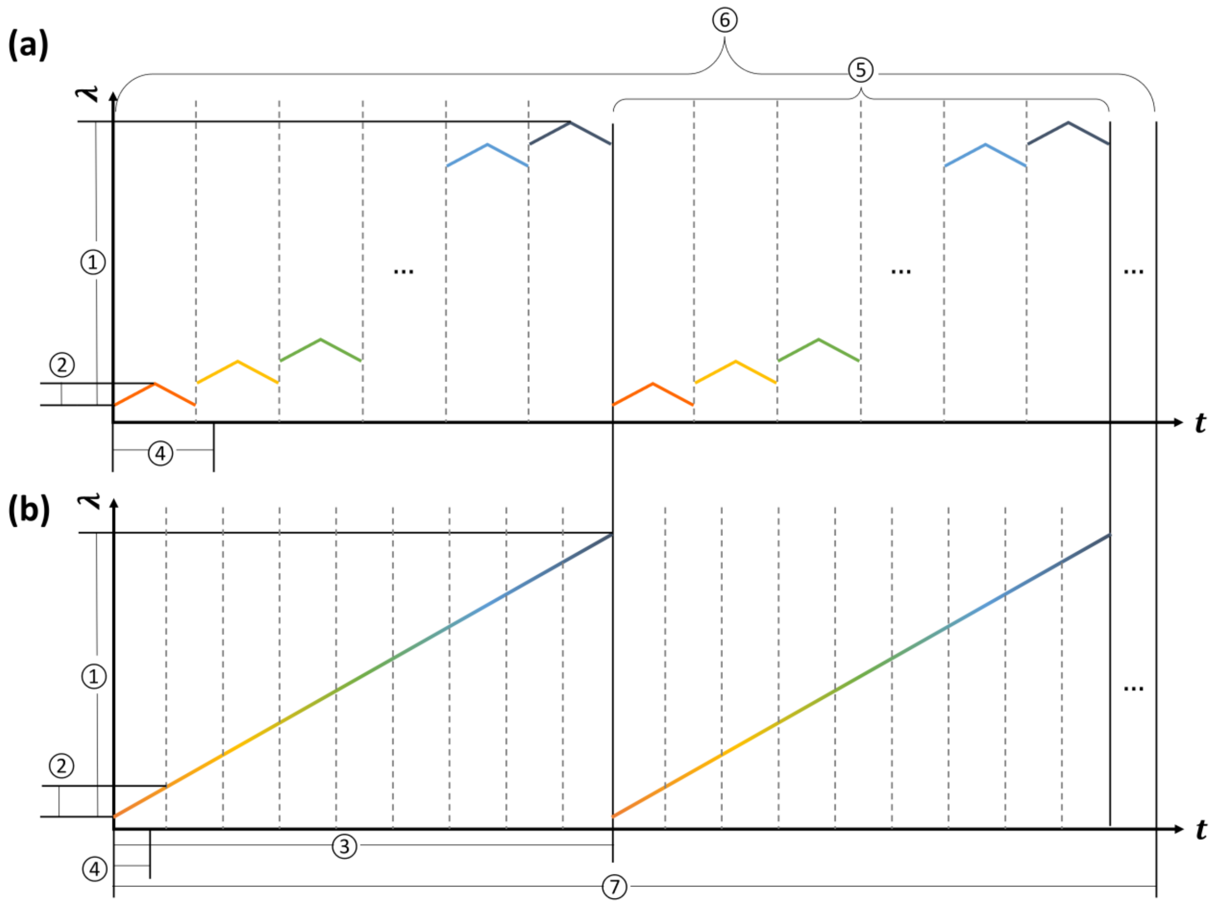

Supplementary Fig. 1. Different spatio-spectral coherent ranging principles based on (a) the flutter-wavelength-swept laser (FWSL) and **(b)** conventional wavelength-swept laser. ① Wavelength sweep bandwidth, i.e., the spatio-spectral field of view (FOV); ② Sampling point interval which corresponds to the flutter-wavelength modulation bandwidth; ③ Fast axis scan rate; ④ Acquisition rate; ⑤ Fast axis pixel number; ⑥ Slow axis pixel number; ⑦ Frame rate. The numbered values for each case are referred to in Supplementary Table 1.

Supplementary Table 1. Specifications of spatio-spectral coherent light detection and ranging (LiDAR).

Content	Laser specification						
	Coherence length	Center Wavelength (nm)	Sweep bandwidth (THz)	Sampling point interval (nm/pt)	Axial resolution (cm)	Acquisition rate (MHz)	Fast axis scan rate (kHz)
Okano et al.[1]	> 150 m (HSL-1, Santec)	1060	11	1.13	0.042	0.3 (N=30)	10
			5	0.412	0.14	0.013 (N=45)	0.3
Qian et al.[2]	> 1 m (akinetic allsemicon ductor programmab le swept laser, Insight Photonics So lutions)	1316	11.4	0.27	0.282	7.6 (N=475)	15.94
Ours (Flutter-wavelength modulation)	> 1.18 km	1535	11.2①	0.0056②	21	0.1④	2③
Ours (Conventional wavelength sweep)	> 1.18 km	1535	11.2①	0.44②	0.27	0.4④ (N=200)	2③

Content	LiDAR system specification					
	Electric bandwidth		Maximum measurable distance (m)	Distance resolution (cm)	Velocity resolution (cm/s)	Frame rate (Hz)
	Photodetector (GHz)	Digitizer (GS/s)				
Okano et al.[1]	1	1	0.25 (*linear tuning period : 35 μs)	N.A.	N.A.	100 (30x100 px)
			12 (*linear tuning period : 800 μs)	N.A.	N.A.	1.5 (45x200 px)
Qian et al.[2]	0.4	0.8	0.33	< 0.08	N.A.	33.2 (475x400 px)
Ours (Flutter-wavelength modulation)	0.4	0.5	264	< 4	< 20	10⑦ (200⑥x45⑤ px)
Ours (Conventional wavelength sweep)	0.4	0.5	1.7	< 4	< 20	44.4⑦ (200⑤x45⑥ px)

Supplementary Note 2. FWSL structure and principles

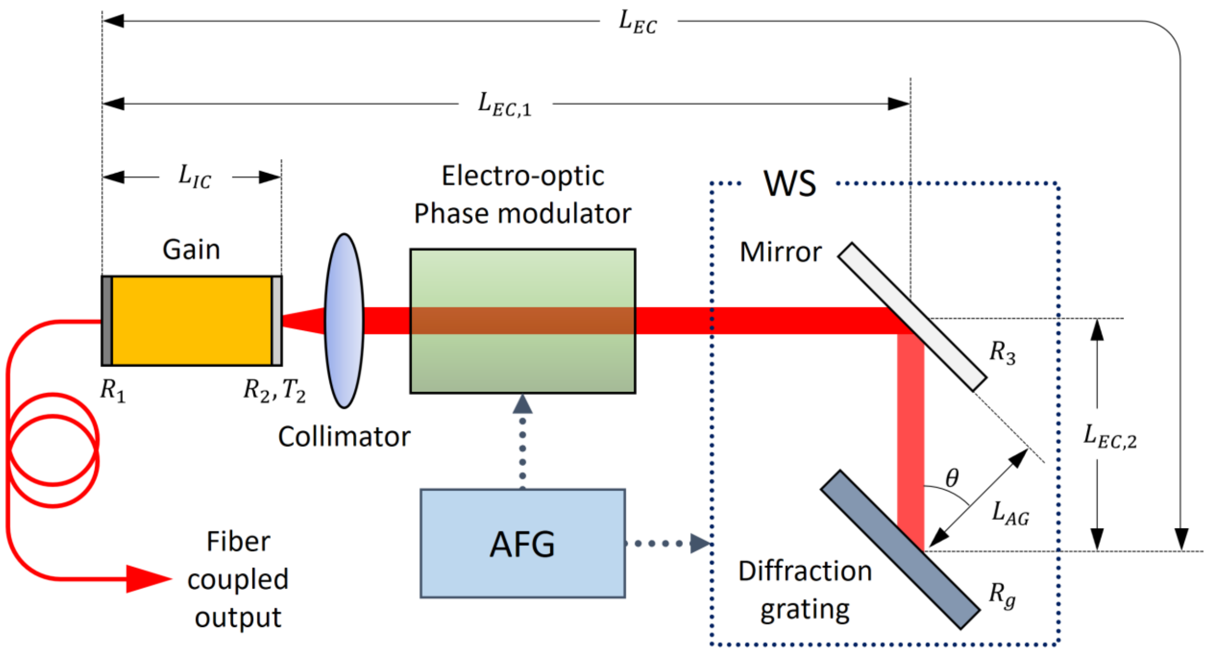

Supplementary Fig. 2. Detailed structure of the FWSL. AFG and WS denote the arbitrary function generator and wavelength selector, respectively.

The detailed structure of the FWSL is shown in Supplementary Fig. 2. The FWSL adopts a Littrow configuration with a tunable reflective filter structure based on a wavelength selector (WS), which consists of a mirror and reflective holographic diffraction grating (1050 grooves/mm). The broadband light generated from the gain is collimated and passed through an electro-optic phase modulator (EOPM), which is for flutter-wavelength modulation. The output wavelength is then selected via the WS by adjusting the angle of light incident on the diffraction grating. The reflected light of the selected wavelength is lased within the cavity from the gain to the WS and emitted through a fiber-coupled output. During the overall operation, the output wavelength sweep via the WS and the flutter-wavelength modulation via the EOPM are synchronized using an electrical control signal from an arbitrary function generator.

The output wavelength of the FWSL is determined by a combination of the cavity spectrum of the gain chip, the longitudinal mode spectrum of the external cavity, and the reflection spectrum of the WS. The internal cavity gain of the gain chip can be expressed as[3,4]:

$$G_{IC} = \frac{G}{\left(1 + G\sqrt{R_1 R_2}\right)^2 + 4G\sqrt{R_1 R_2}\sin^2\theta} \quad (1)$$

where G denotes the single pass gain, R_1, R_2 indicates the reflectance of both facets of the gain chip, and θ refers to the phase difference that can be expressed as $\theta = 4\pi L_{IC}/\lambda$, where L_{IC} and λ denote the internal cavity length and wavelength, respectively[4]. The external cavity gain is expressed as[4]:

$$G_{EC} = \frac{T_2}{\left[1 - G\sqrt{R_1}T_2 R_3\sqrt{R_4}\right]^2 + 4G\sqrt{R_1}T_2 R_3\sqrt{R_4}\sin^2\delta} \quad (2)$$

where R_3 and R_4 indicate the reflectance of the angle scanner and diffraction grating, respectively, and δ denotes the phase difference, which has a relation of $\theta = 4\pi L_{EC}/\lambda$ with external cavity length, L_{EC}. The reflectance of the diffraction grating can be expressed as[4]:

$$R_4 = R_G\left(\frac{\sin(N\phi/2)}{N\sin(\phi/2)}\right)^2 \quad (3)$$

where R_{g} denotes the peak reflectance of the grating, N corresponds to the illuminated groove number of the diffraction grating, and ϕ symbolizes the phase difference, which can be calculated by $\phi = 2m\pi\lambda_0/\lambda$, where λ_0 indicates the feedback wavelength of the grating. The gain spectrum of the FWSL is determined by the product of the above factors and is expressed as[4]:

$$G_{\mathrm{net}} = R_4 G_{IC} G_{EC} \quad (4)$$

For simplicity, the losses of the collimator and the EOPM are ignored.

Supplementary Fig. 3 shows the structural configuration of the FWSL cavity mode. The cavity configuration parameters used in the simulations are listed in Supplementary Table 2. Based on these simulations, three identical lasers were fabricated and operated well as intended, demonstrating the designed features of the FWSL.

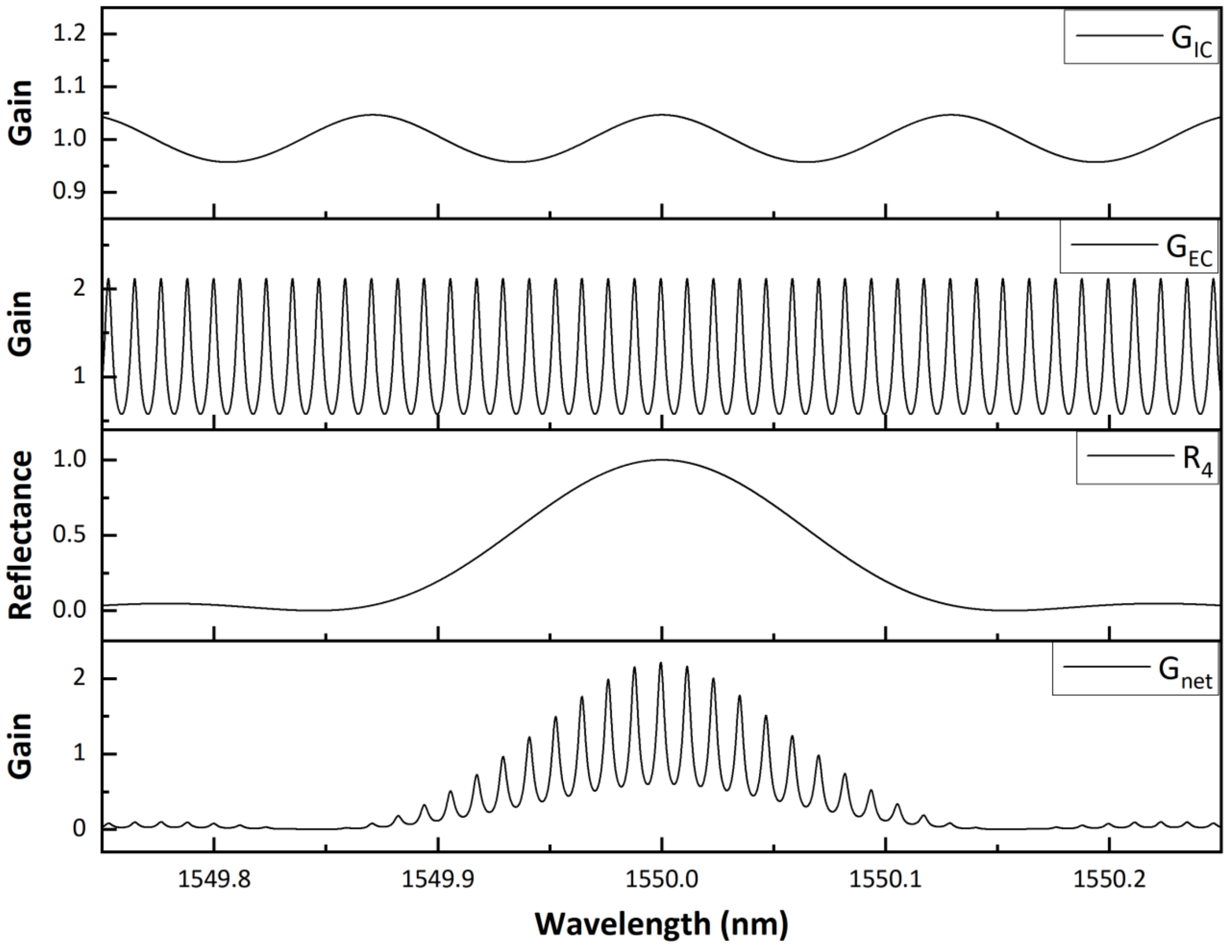

Supplementary Fig. 3. Simulation results of the cavity mode structure of the FWSL.

Supplementary Table 2. Parameters and values for the cavity spectrum simulation.

Parameter	L_{IC} (mm)	L_{EC} (mm)	G	R_1	R_2 (T_2)	R_3	R_g	λ_g (nm)	N
Value	4.65	50.1	1	0.1	0.005 (0.995)	1	1	1550	10000

Supplementary Note 3. Principle of wavelength change

It has previously been confirmed that the output wavelength of the FWSL is determined by a complex spectrum of the internal and external cavities, and each cavity gain is derived from the cavity length. The external cavity, which exhibits a relatively high gain, primarily influences wavelength selection. In other words, the output wavelength can be changed by adjusting the length of the external cavity of the FWSL[5]. In addition, the output wavelength could be changed by varying the angle of light incident on the diffraction grating[3].

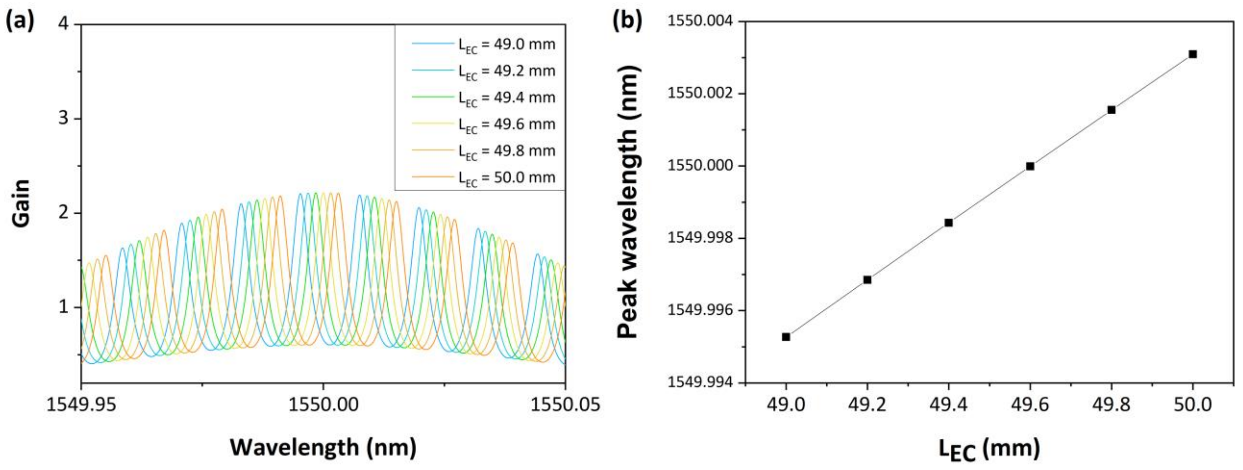

Supplementary Fig. 4. (a) Simulation results of G_{EC} according to L_{EC} change; (**b)** Resulting peak wavelength change.

The FWSL utilizes both techniques for wavelength changes. First, the flutter-wavelength modulation was achieved by controlling the external cavity length through the EOPM at each fixed angle of incident light on the diffraction grating. Supplementary Fig. 4 illustrates the simulation results of the different gain spectrum when the external cavity length was changed. The remaining parameters, except L_{EC}, used the same values as those listed in Supplementary Table 2.

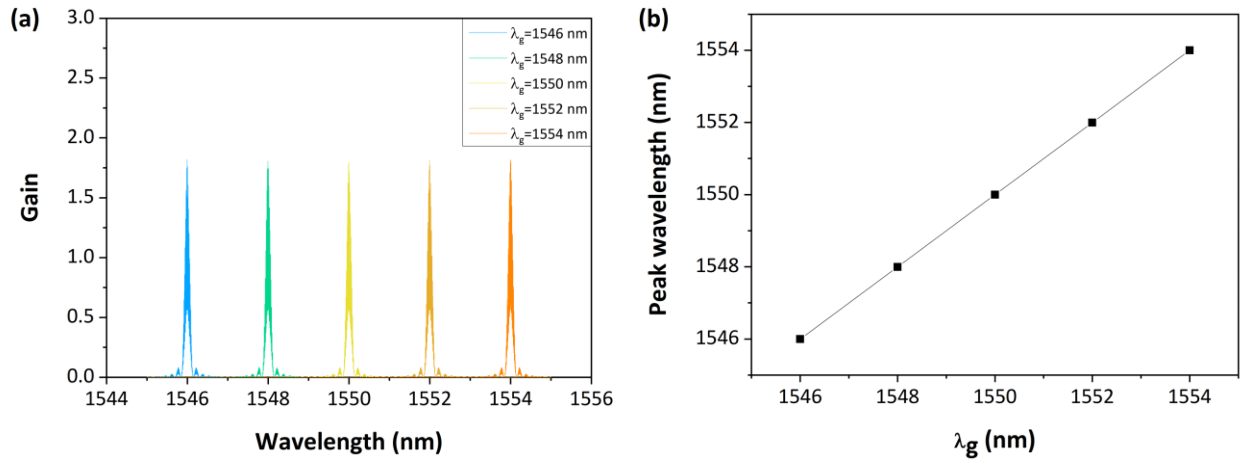

Supplementary Fig. 5. (a) Simulation results of G_{EC} based on the change in λ_g; **(b)** Resulting peak wavelength change.

Next, a wavelength sweep was implemented using the WS by changing the angle of light incident on the diffraction grating. In the Littrow configuration, the first-order feedback wavelength of the diffraction grating can be expressed as[3]:

$$\lambda_g = 2d \sin\theta \tag{5}$$

where d and θ denote the grating period and the angle of light incident on the diffraction grating, respectively. Based on the gain spectrum of the external cavity (Eq. 2) along with the effect of the diffraction grating, only external cavity modes of a particular order are selected and survive, depending on the angle of light incident on the diffraction grating. Supplementary Fig. 5 presents the simulation results of the different gain spectrum when λ_g changes. For the simulation, the remaining parameters, except for λ_g, had the same values as those listed in Supplementary Table 2.

Supplementary Note 4. Wavelength modulation bandwidth

The mode-hop-free wavelength modulation range through a variable external cavity length is limited by the free spectral range (FSR) of the cavity as follows[3]:

$$\left|v_{EC} - v_g\right| \leqq \frac{1}{2} FSR \tag{6}$$

where v_{EC} and v_g denote the peak external cavity mode frequencies within the diffraction grating reflection spectrum region and the peak reflection spectrum of the diffraction grating, respectively. FSR is determined as $FSR = \frac{c}{2L_{EC}}$, where c refers to the speed of light in a vacuum.

Owing to the structural characteristics of the FWSL, the external cavity length changed when the wavelength was swept through the WS, as shown in Supplementary Fig. 2. The external cavity length, as shown in Supplementary Fig. 2, can be expressed as:

$$L_{EC} = L_{EC,1} + L_{EC,2} = L_{EC,1} + \frac{L_{AG}}{\cos\theta} \tag{7}$$

Accordingly, FSR can be expressed as:

$$FSR(\lambda) = \frac{c}{L_{EC,1} + \frac{L_{AG}}{cos\left\{sin^{-1}\left(\frac{1}{2d\lambda}\right)\right\}}} \tag{8}$$

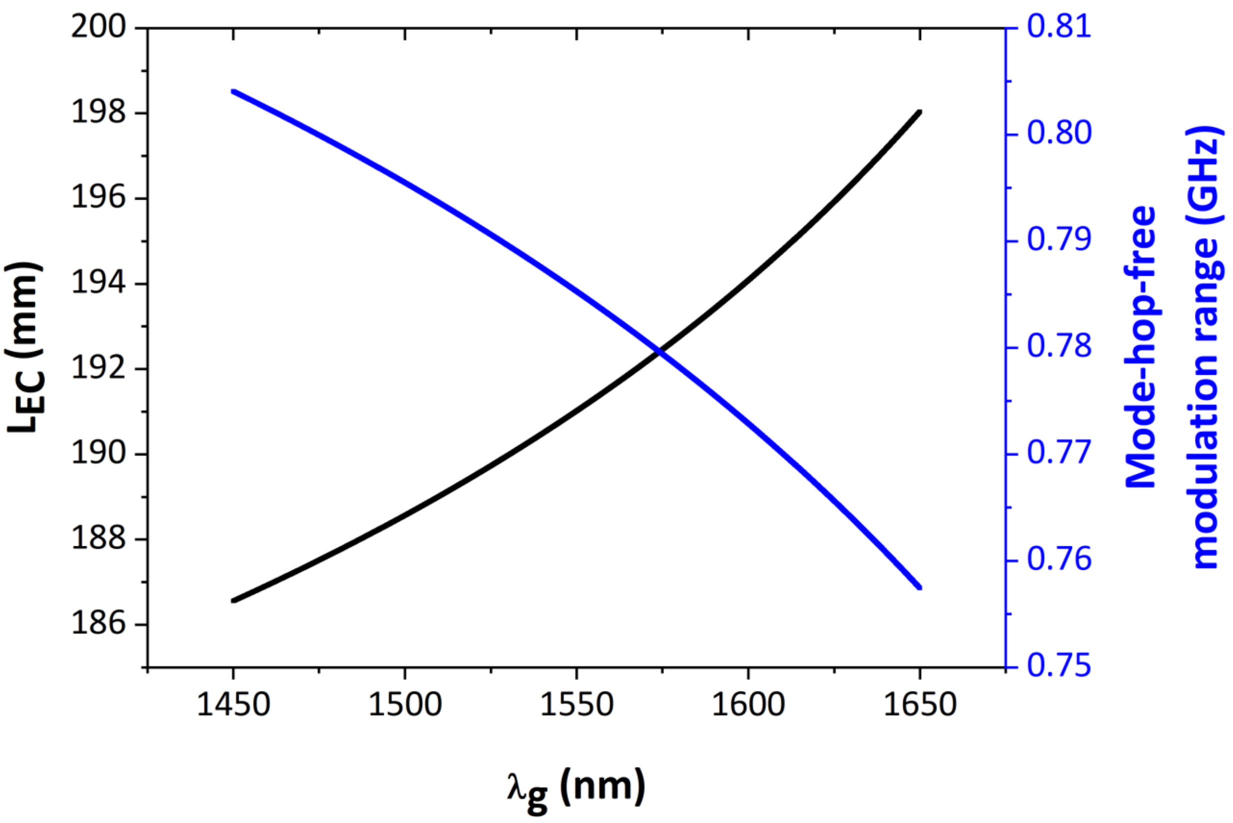

Supplementary Fig. 6. Simulation results of differences in L_{EC} and mode-hop-free modulation range based on the output wavelength λ_g of the FWSL.

L_{EC} and mode-hop free modulation range based on the output wavelength of the FWSL were simulated using the specifications of the fabricated FWSL—$L_{EC,1} \simeq 150\ mm$, $L_{AG} \simeq 25\ mm$, $d = 1050\ \text{grooves/mm}$—are shown in Supplementary Fig. 6. As shown in Supplementary Fig. 2, the FWSL was designed such that L_{EC} increased with the reflected wavelength of the WS. According to the present architecture, the FWSL lengthens L_{EC} by approximately 11 mm, whereas the output wavelength sweeps from 1450 to 1650 nm. An increase in L_{EC} decreases FSR, and the mode-hop-free modulation range also decreases, according to Eq. 6.

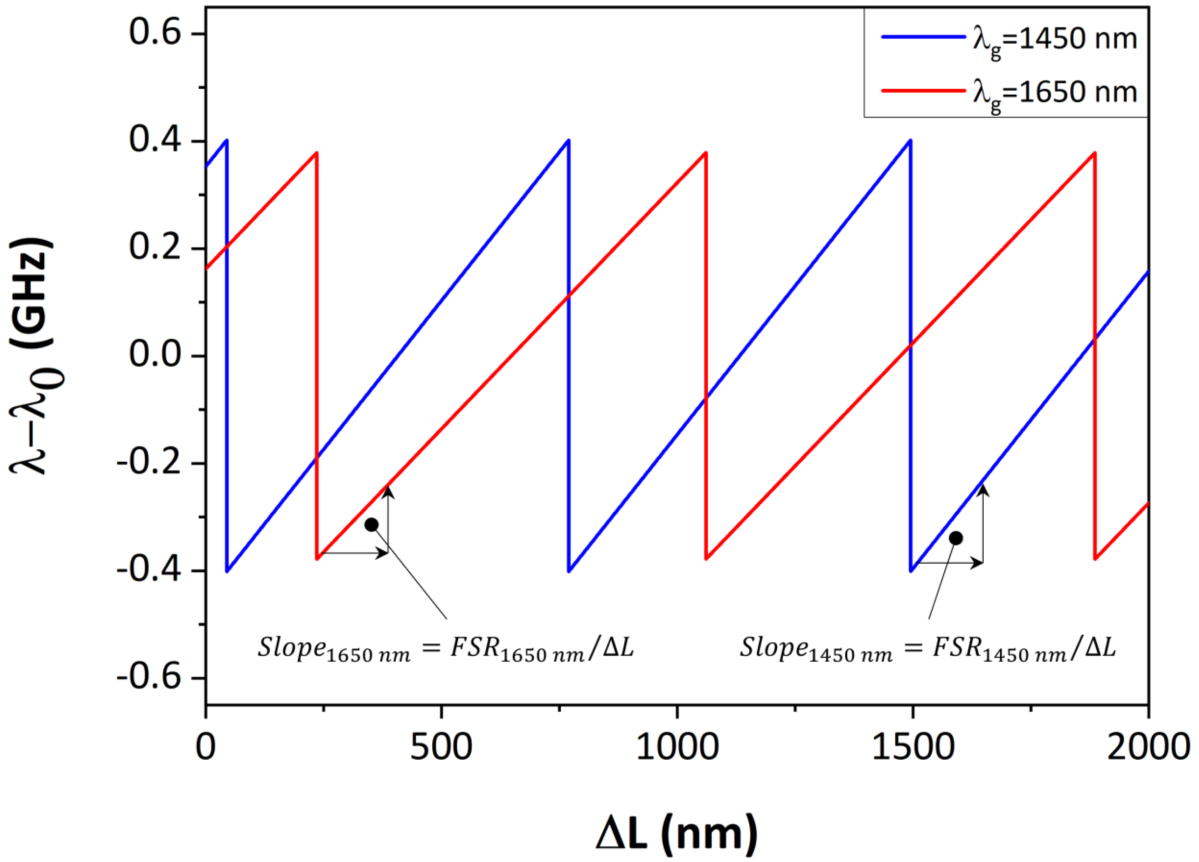

Supplementary Fig. 7. Simulation results of the output wavelength change based on the same cavity length change at different λ_g.

Supplementary Table 3. Calculation results of FSR, $Slope$, and relative ratios of $Slope$ according to the wavelength.

Wavelength [nm]	1480	1500	1520	1540	1560	1580	1600
FSR [GHz]	0.7991	0.7955	0.7916	0.7875	0.783	0.7782	0.7729
Slope [GHz/nm]	0.00108	0.001061	0.001042	0.001023	0.001004	0.000985	0.000966
Norm. slope [%]	100	98.235	96.481	94.732	92.984	91.233	89.486

Supplementary Fig. 7 shows the simulation results based on Eq. 4, which shows the change in the output wavelength when the cavity length is varied. The change in the output wavelength ($Slope$) according to the optical path difference can be expressed as:

$$Slope(\lambda) = \frac{2 \cdot FSR(\lambda)}{\lambda} \quad (9)$$

Supplementary Table 3 contains the results of the calculated FSR, $Slope$, and the relative ratio of $Slope$ for each wavelength. The FSR values derived from Supplementary Fig. 6 were used for the calculation. From the calculations, the change in the output wavelength ($Slope$) according to the varying cavity length tended to decrease by approximately 89% as the center wavelength increased.

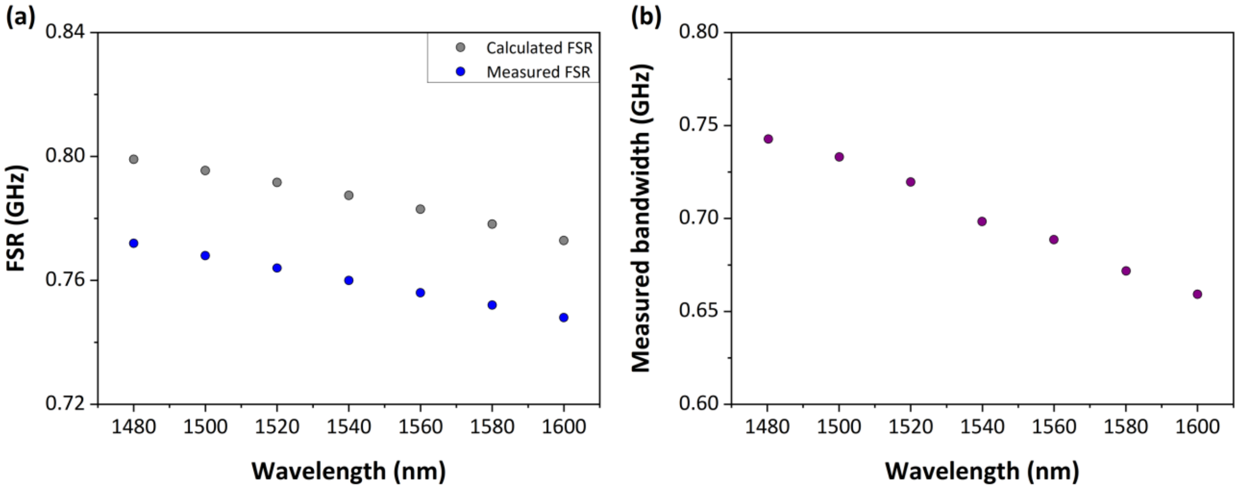

Supplementary Fig. 8. (a) Calculated and measured $\boldsymbol{FSR}$ based on the wavelength; **(b)** Measured wavelength modulation bandwidth based on the wavelength with respect to the same electro-optic phase modulator (EOPM) operation.

Supplementary Fig. 8a shows the calculated FSR and measured FSR of the FWSL. It is evident from the figure that the calculated and measured FSR changed equally depending on the wavelength, with similar reduction rates. The difference between the calculated and measured values was expected to occur during the component assembly and alignment processes of the FWSL.

Supplementary Fig. 8b shows the measured modulation bandwidths at each wavelength. As confirmed by Eq. 9 and Supplementary Table 3, the actual modulation bandwidth tended to decrease as the selected wavelength increased. Supplementary Table 4 depicts the measured modulation bandwidth for each wavelength and the standard relative ratio. In particular, the Norm. modulation bandwidth in Supplementary Table 4 exhibited a tendency similar to that of the Norm. slope and the theoretical analysis values in Supplementary Table 3.

Supplementary Table 4. Measured modulation bandwidth and relative ratios based on the wavelength.

Wavelength [nm]	1480	1500	1520	1540	1560	1580	1600
Modulation bandwidth [GHz]	0.74273	0.73303	0.71968	0.69843	0.68858	0.67177	0.65923
Norm. modulation bandwidth [%]	100	98.694	96.896	94.035	92.709	90.446	88.757

Supplementary Note 5. Mode hopping

Previously, the mode-hop-free condition in the external cavity during wavelength modulation through EOPM was confirmed using Eq. 6. However, mode hopping may occur if the initial state has a sufficiently large difference between the external cavity mode and central reflection wavelength of the diffraction grating. Furthermore, the unexpected mode hopping phenomenon is believed to be caused by asymmetric nonlinear gain[6–9]. However, such mode hopping phenomenon could hardly be observed in the actual experimental process during the two years of the expected lifetime of the fabricated FWSL.

During the wavelength sweep through the WS, mode hopping inevitably occurs because the mode order of the external cavity changes. Mode hopping can be suppressed using a pivot-based external cavity structure[10] or quasi-phase continuous tuning[11] that matches the reflected wavelength of the diffraction grating and external cavity mode. However, in the proposed FWSL, the WS and EOPM operate sequentially; therefore, a fixed-order cavity mode is ensured during flutter-wavelength modulation through the EOPM. In addition, the measured modulation bandwidth (Supplementary Fig. 8b, Supplementary Table 4) was within the maximum mode-hop-free modulation range (Supplementary Fig. 8a, Supplementary Table 3), and hysteresis was observed[6–9]. Mode hopping during the EOPM driving process is expected to occur rarely, which can be fatal in axial coherent ranging.

Supplementary Note 6. Optical linewidth of the FWSL

The proposed FWSL is a wavelength-swept laser used for long-range coherent LiDAR. Among the primary factors required for a laser source in remote sensing, a long coherence length is the most fundamental. The optical linewidth of the FWSL was measured to estimate the coherence length. A self-heterodyne interferometer[12,13] with a 10-km fiber delay line was used for the measurement. An electro-optic modulator was used as a frequency shifter driven by a 25 MHz sine function. The center wavelength of the FWSL was set to 1535 nm, and the output spectrum was measured using a spectrum analyzer as shown in Supplementary Fig. 9. The half-width at half-maximum of the Lorentz fitting of the measured spectrum was 81 kHz. The corresponding coherence length was approximately 1.2 km[12,13].

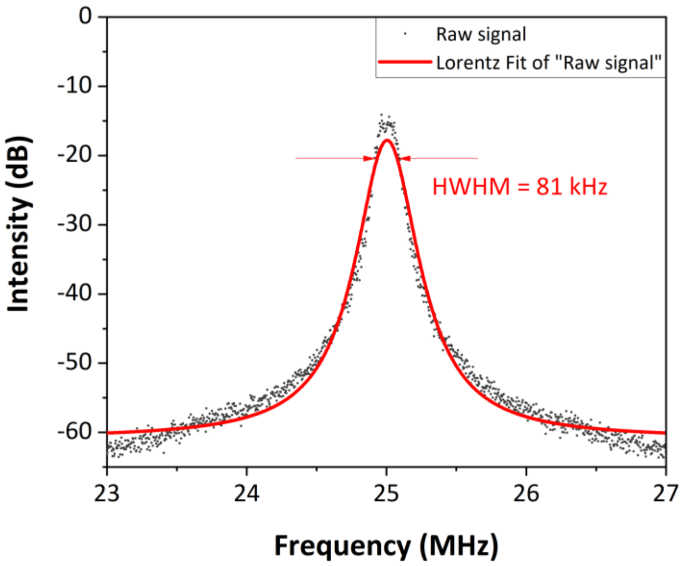

Supplementary Fig. 9. Measured linewidth with the Lorentz fit. HWHM denotes half-width at half-maximum.

Supplementary Note 7. Flutter-wavelength modulation characteristics

A linear change in the optical frequency, that is, the wavelength, is essential for coherent ranging to obtain accurate measurements. Commercial wavelength-swept lasers typically require a separate k-linearization process[14] to ensure sweep linearity. However, we did not include an additional k-linearization process in our proposed system because the FWSL ensures high linearity during wavelength modulation. The relative optical frequency was measured to evaluate the k-linearity during flutter-wavelength modulation. A fiber-based 4 × 4 Mach–Zehnder interferometer with a 10 m fiber delay line was designed for the measurement. Two interference signals with phase differences of 90° were balanced detected. The original interference signals shown in Supplementary Fig. 11, 12, and 13 represent one of the two collected interference signals. The phase change of the laser source was extracted via I-Q demodulation and converted into a relative optical frequency unit based on the following relation[15,16]:

$$I_n = A_n \cos\left(2k\Delta z + \emptyset_n\right) \tag{10}$$

where I_n, A_n, k, Δz, and $\emptyset$ denote the interference signal, signal amplitude, wavenumber, optical path difference, and phase, respectively.

First, the flutter-wavelength modulation results were measured by varying the amplitude of the EOPM modulation signal. A triangular waveform with a 100 kHz modulation rate was fixed, while the center wavelength was 1535 nm. The flutter-wavelength modulation results shown in Supplementary Fig. 10 indicate that the relative optical frequency changed equally along the applied modulation signal and that the modulation bandwidth increased proportionally with the amplitude of the modulation signal.

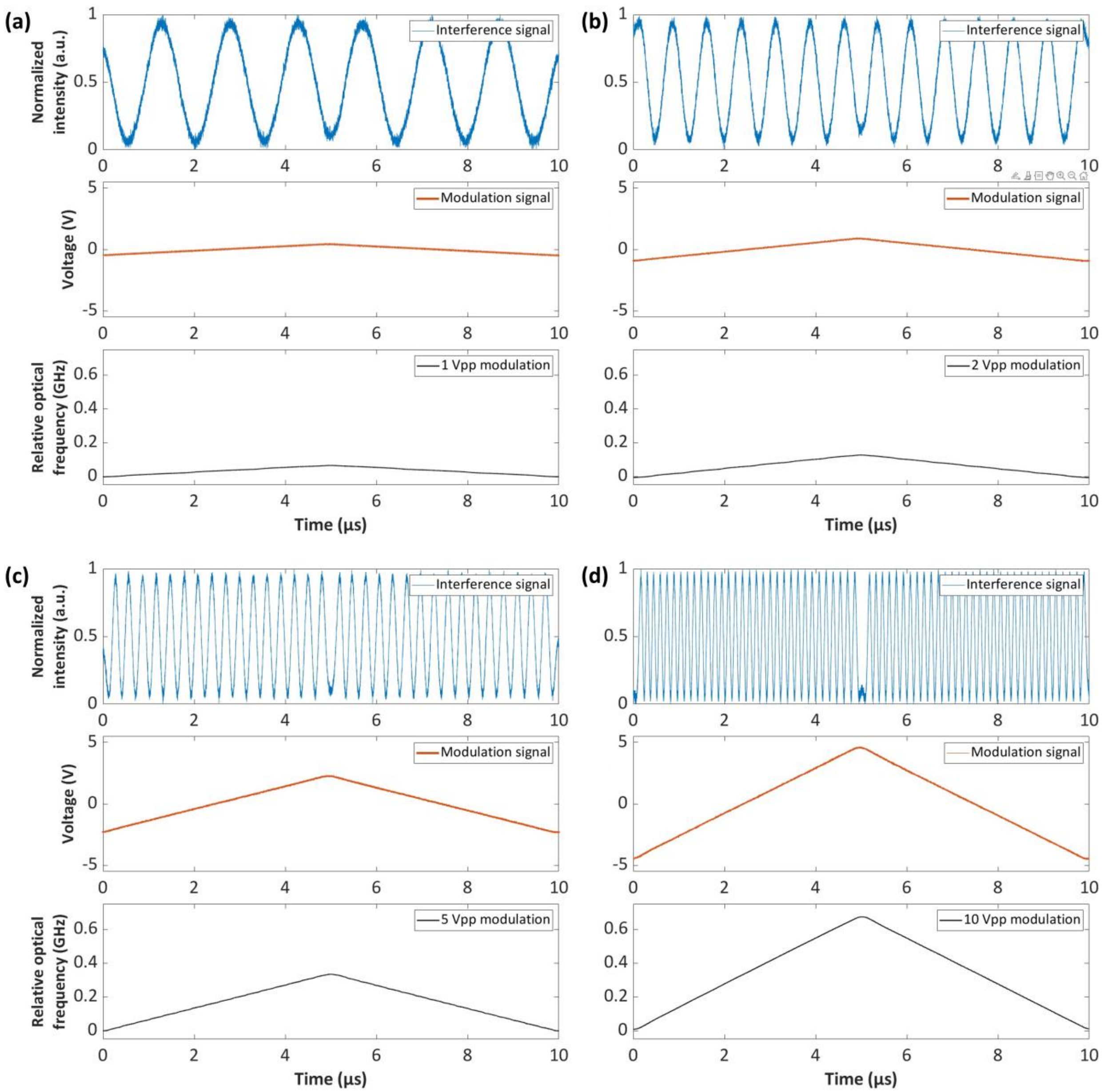

Supplementary Fig. 10. Flutter-wavelength characteristics based on the modulation voltage. Raw interference signal of the single modulation period, monitoring signal from voltage amplifier (attenuated to 5% of the actual modulation signal output), and relative optical frequency decomposed from the interference signal. Flutter-wavelength modulation results from a 100 kHz triangular waveform from an arbitrary function generator with modulation amplitude of **(a)** 1 Vpp, **(b)** 2 Vpp, **(c)** 5 Vpp, and **(d)** 10 Vpp.

Subsequently, the same measurements were performed by varying the speed of the EOPM modulation signals. A triangular waveform with a modulation amplitude of 10 Vpp was fixed, and the center wavelength was set to 1535 nm. The measured flutter-wavelength modulation results are shown in Supplementary Fig. 11, indicating the equal behavior of the optical

frequency relative to the modulation signal. However, when the modulation rate reached 200 kHz, the change in the central vertex section of the modulation signal became blunt. Accordingly, the optical frequency change appeared to be the same in the corresponding areas, indicating that the response of the voltage amplifier was insufficient for high-speed and high-amplitude signals.

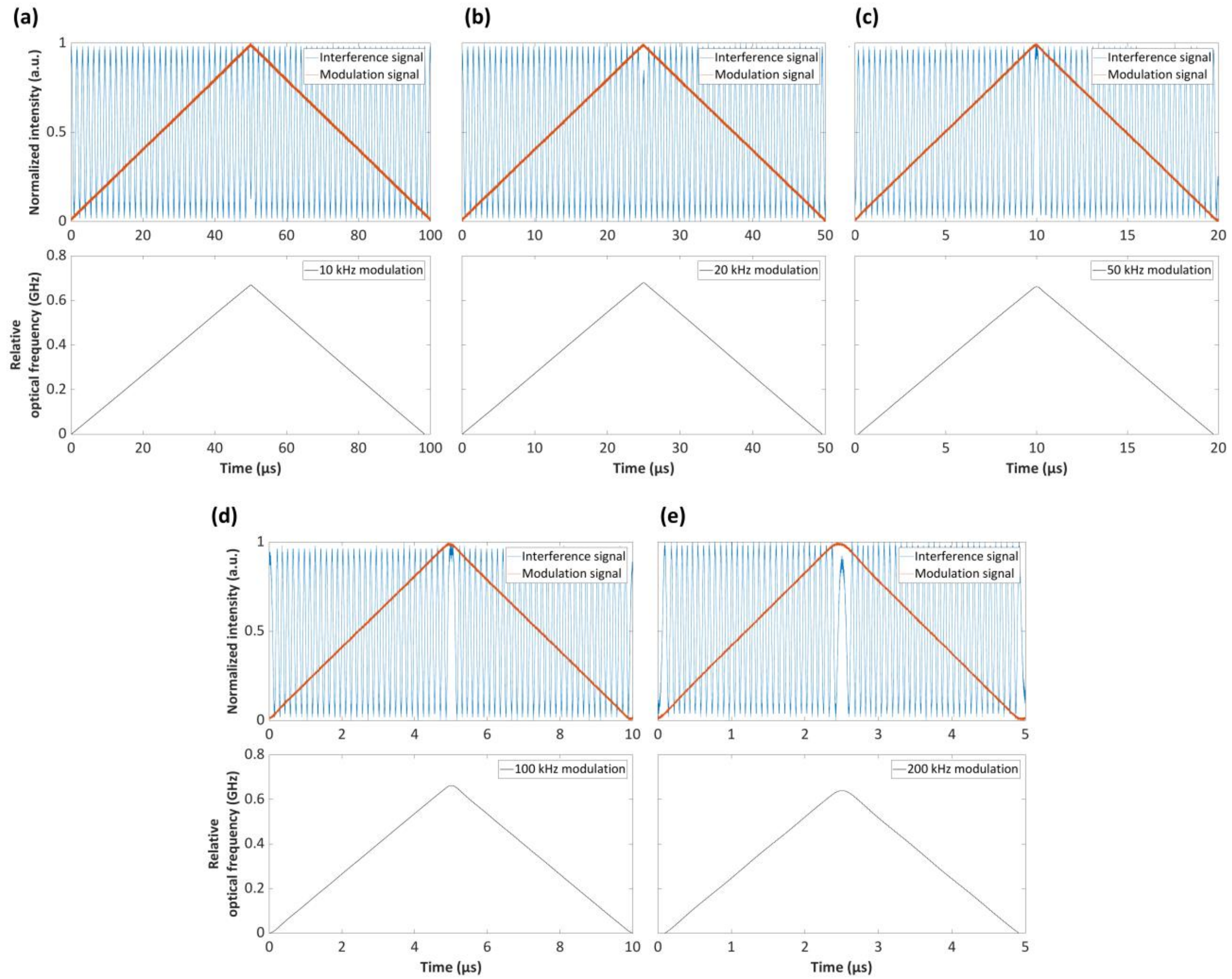

Supplementary Fig. 11. Flutter-wavelength characteristics according to the modulation rate. The raw interference signal of a single modulation period overlapped with the monitoring signal from the voltage amplifier and the relative optical frequency change. Flutter-wavelength modulation results from a 10 Vpp triangular waveform generated using an arbitrary function generator with modulation rates of **(a)** 10 kHz, **(b)** 20 kHz, **(c)** 50 kHz, **(d)** 100 kHz, and **(e)** 200 kHz.

Based on the analysis of the flutter-wavelength modulation characteristics under various conditions, a triangular waveform with an amplitude of 200 Vpp and a rate of 100 kHz was fixed for axial coherent ranging throughout the experiment. The linearity of the optical frequency

change was evaluated under the specified modulation conditions. From the linear fitting results in Supplementary Fig. 12c and d, R-squared values of 0.99997 and 0.99987 were confirmed for each linear variable period for the up- and down-chirps, respectively. The high linearity of the FWSL facilitates coherent ranging without additional k-linearization.

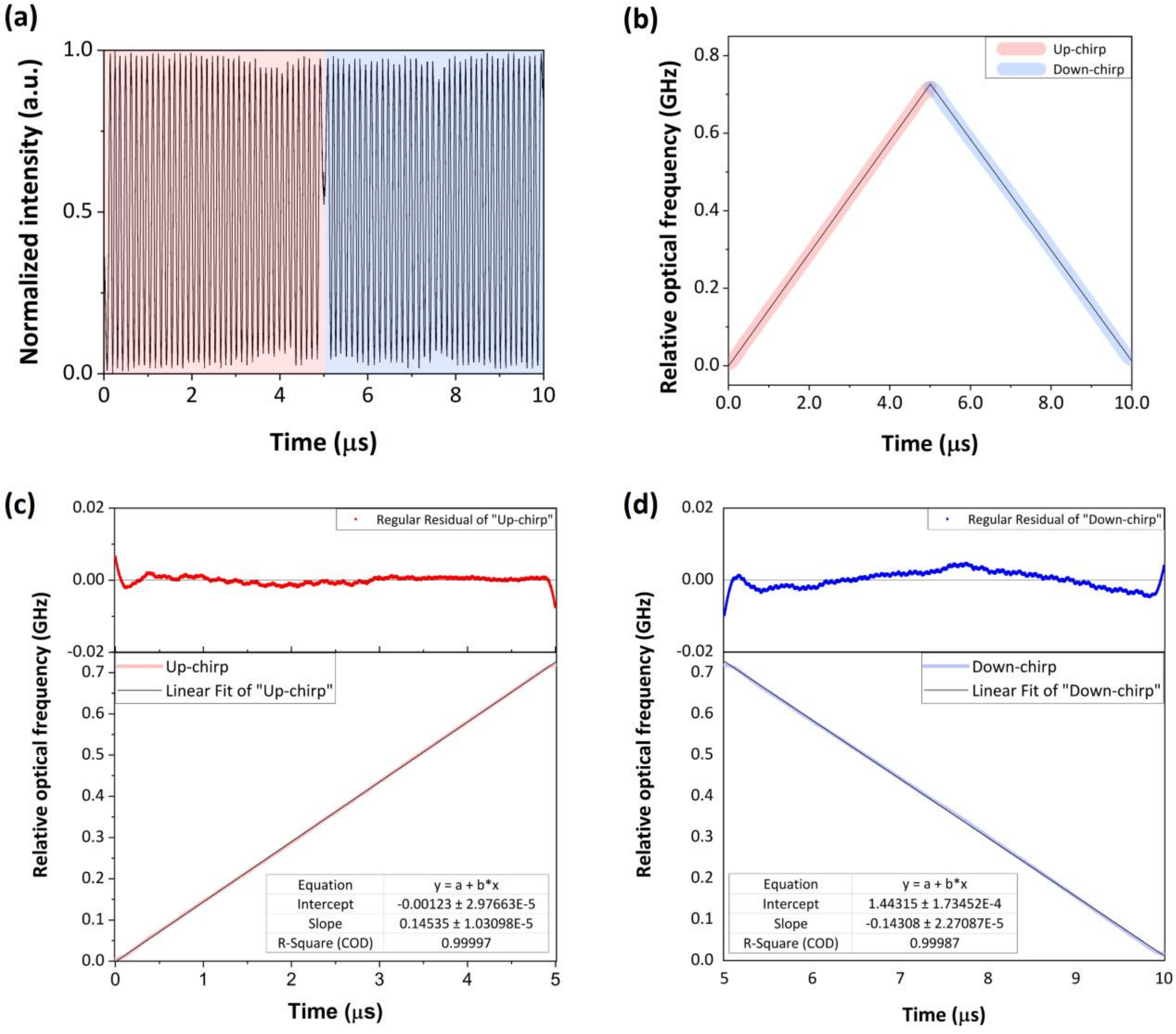

Supplementary Fig. 12. Linearity evaluation of flutter-wavelength modulation. (a) Raw interference signal; **(b)** Relative optical frequency behavior; Linear analysis including a regular residual plot of **(c)** up-chirp and **(d)** down-chirp.

Supplementary Note 8. Spectral bandwidth of the FWSL

The spectral bandwidth of the FWSL is shown in Fig. 2e, which was measured by discretely sweeping the wavelength. We conducted additional measurements to demonstrate the capability of a continuous wavelength sweep of the FWSL by continuously varying the WS. The output spectrum was measured in the maximum-hold mode of an optical spectrum analyzer. Supplementary Fig. 13 shows the continuous wavelength change for a spectral bandwidth of 160 nm.

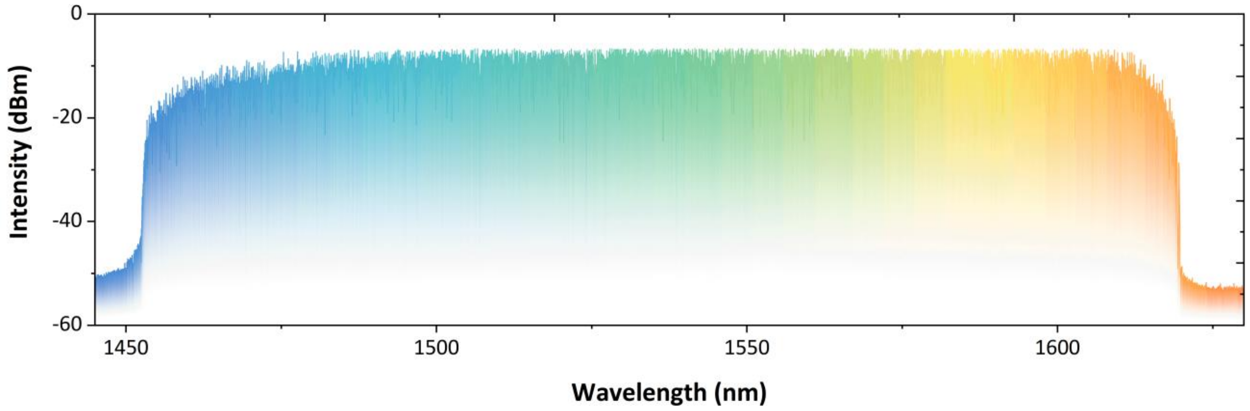

Supplementary Fig. 13. Continuous wavelength sweep bandwidth of the FWSL.

Supplementary Note 9. Axial coherent ranging precision

A one-dimensional (1D) coherent measurement of a static target was performed to analyze the accuracy and precision of the axial coherent ranging of the proposed system. The experimental setup shown in Fig. 2a is used the same, and a retroreflective tape was used as the target. A total of 1000 measurements were collected at a single axial point without spatial scanning. EOPM was modulated in the fixed condition described above, and both the acousto-optic deflector (AOD) and WS were set to the center acoustic frequency and center wavelength, respectively. The target distance was measured from 1 to 25 m at 2-m intervals with respect to the transmission grating (TG). As shown in Supplementary Fig. 14a, coherent ranging was conducted well up to a distance of 25 m, which showed an R-squared value of 0.9999 for the average distance. It is observed that the error value tended to increase as the distance increased. Relatively high error values at long distances were assumed because of the working distance of the optical system. Supplementary Fig. 14b presents the standard deviation (SD) of 1000 measurement results, indicating the distance precision of the proposed system. The SD tended to increase as the distance increased; however, all the values were recorded below 0.04 m. The results demonstrate that the proposed FWSL enables more precise coherent ranging than the inherent axial resolution limited by the FWSL over long distances.

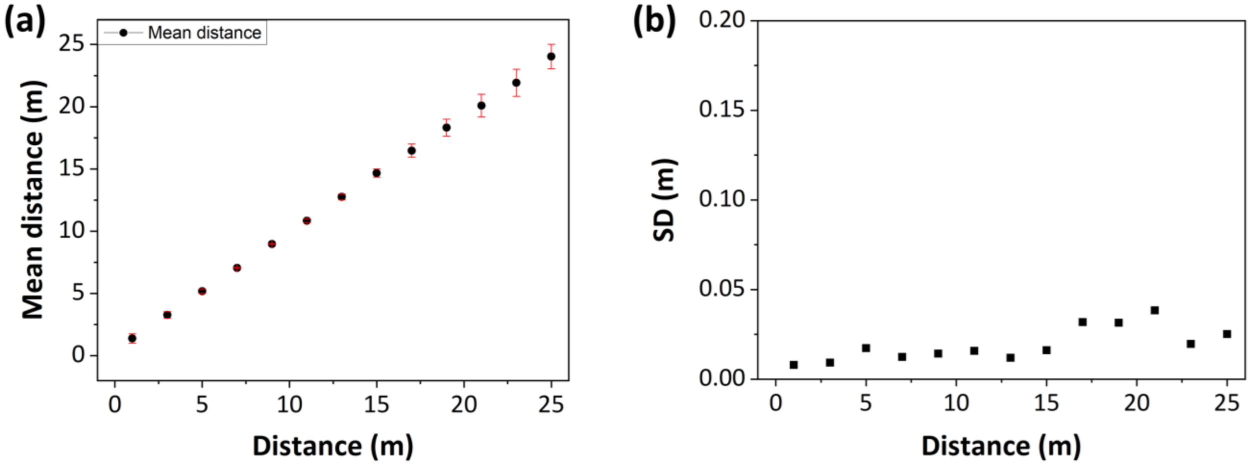

Supplementary Fig. 14. Axial coherent ranging results. (a) Measured mean distance at different target distances with error; **(b)** Standard deviation (SD) of the measurements at different target distances.

Supplementary Note 10. Coherent measurements on a rotating disk

The rotating disk used to demonstrate the velocity measurement of the proposed system is shown in Supplementary Fig. 15a. A retroreflective tape is attached to the surface of the disk, of which the radius was 0.125 m. The disk was rotated at 770 rpm, and the corresponding tangential velocity was 10.08 m/s. The rotating disk was located at a distance of 1 m from the TG.

The theoretical velocity along the optical axis based on the horizontal field of view (FOV) is shown in Supplementary Fig. 15c. The theoretical velocity was calculated using the following equation[17]:

$$v = v_t \sin(\theta + \theta_t)$$
$$\theta_t = \sin^{-1}[(2R/D)\sin\theta] \tag{11}$$

where R is given as $R = {}^{r}/_{\cos\theta}$. The parameters of the relationships are presented in Supplementary Fig. 15b. The region of interest (ROI) was set to include the center of the disk at normal incidence of the beam, where the Doppler shift did not occur because of the absence of velocity components along the optical axis. The ROI is depicted as a red box in the photograph of the rotating disk (Supplementary Fig. 15a).

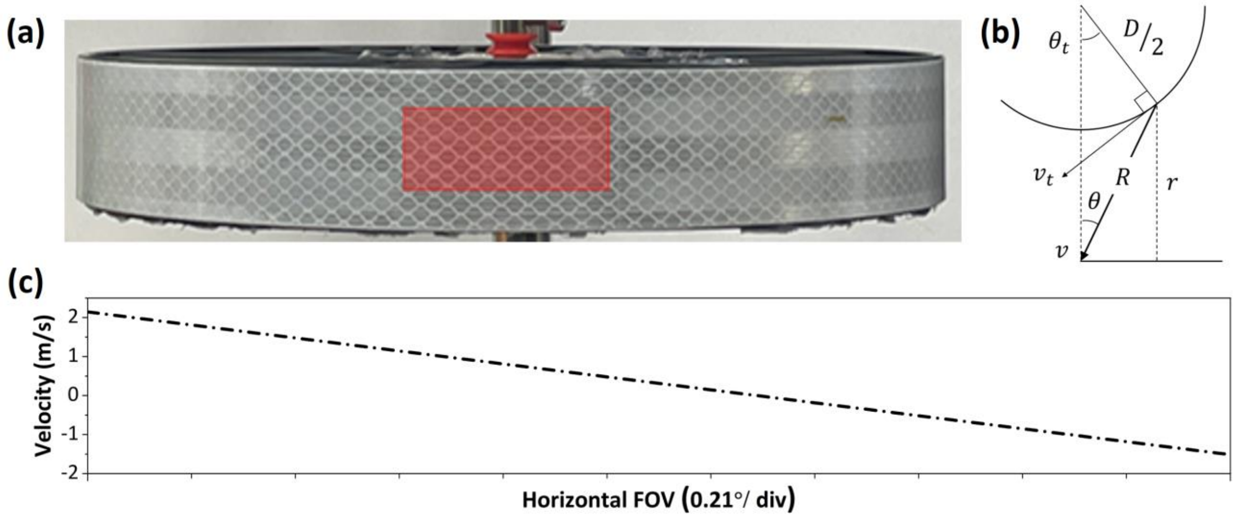

Supplementary Fig. 15. Details of coherent ranging on the rotating disk. (a) Photograph of the rotating disk; **(b)** Parameters of the beam (arrow) and disk (circular arc); **(c)** Theoretical velocity of the disk for the region of interest based on the horizontal FOV.

Supplementary Note 11. 4D coherent ranging in an artificial fog environment

Scene D in Supplementary Fig. 16 was newly designed for real-time 4D imaging verification in a simulated fog environment. Against a retroreflective box at 4.4 m in the background, the rail was located at 3.5 m, and the tree was located at 3.8 m, which are the same objects used in Scenes A and C. To create an artificial fog environment, a studio that could cover the rail was established, and an air distributor was installed on the ceiling of the studio to spray the smog uniformly. A photodiode sensor was placed in the background to measure transmittance. The transmittance was calculated by detecting the optical power of the 1550 nm center wavelength reference laser light passing through the studio. A fiber collimator used to couple the reference laser light into free space was placed parallel to our optical system, and the collimated beam was aligned in a straightforward manner to the photodiode sensor.

The ROI in Supplementary Fig. 16a was imaged at 200 × 45 pixels in real time under scanning conditions of 6.9° × 1.63° (H × V) with an acquisition rate of 100 kHz and a fast axis scan rate of 2 kHz. The final output power of the system was boosted to 20 mW using a booster optical amplifier (Thorlabs, Newton, NJ, USA). A full video of the measurements with photographs and the transmittance of the studio environment is shown in Supplementary Video 2. It is obvious that the 3D distance and velocity of both stationary and moving objects are accurately measured simultaneously regardless of the environment, when comparing the 100% transmittance situation (Supplementary Fig. 16a) and the low transmittance situation (Supplementary Fig. 16b–d). We suggest that the results further demonstrate the robustness of the proposed LiDAR system relative to its powerful and effective 4D imaging.

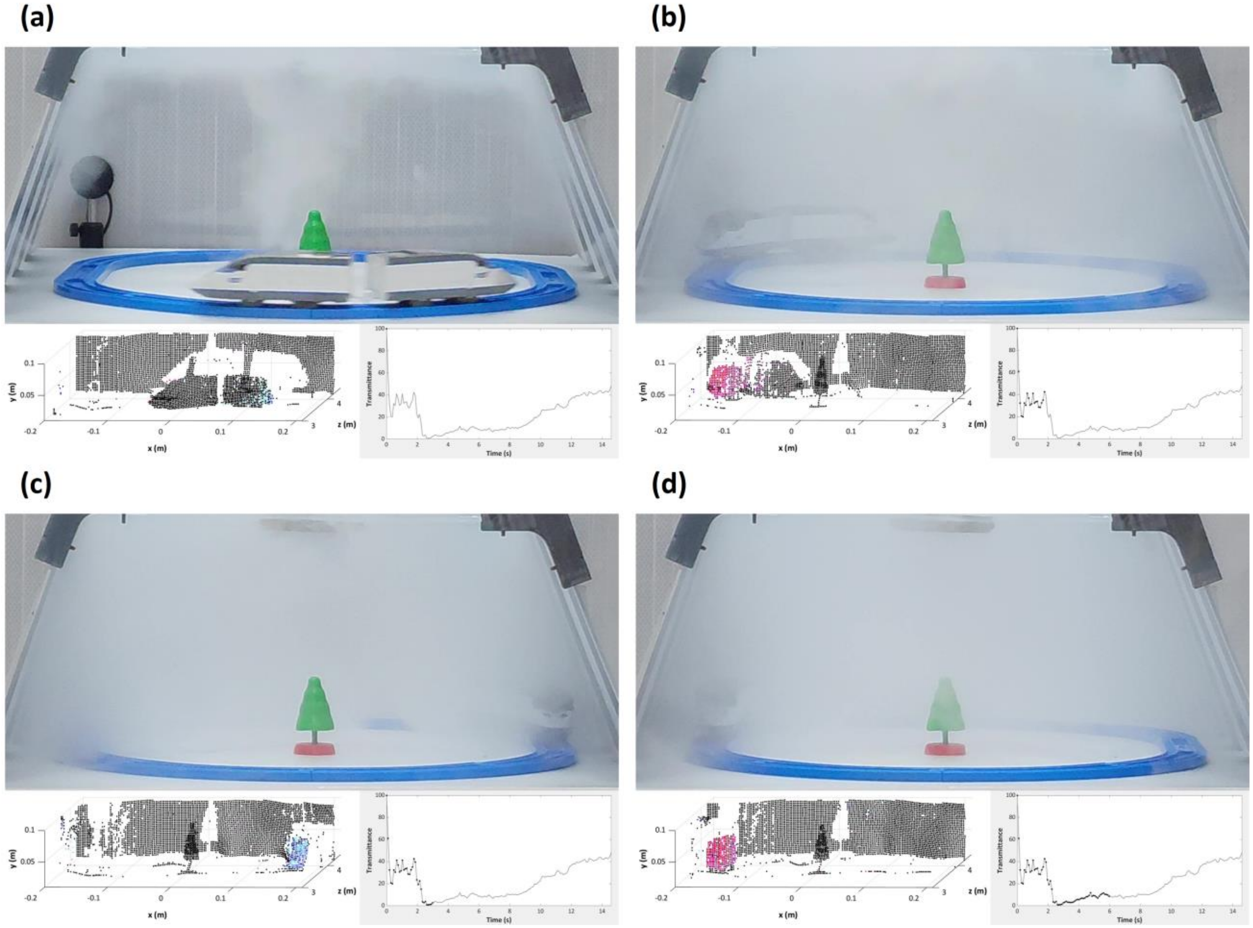

Supplementary Fig. 16. Real-time 4D coherent ranging in a fog environment. (a–d) Photographs of the studio environment, including the 4D distance and velocity image (lower left side) of Scene D sized 200 × 45 pixels at a frame rate of 10 Hz and the transmittance at a given environment (lower right side). Four individual frames were captured from the full video of 14.8 s, which is provided in Supplementary Video 2.

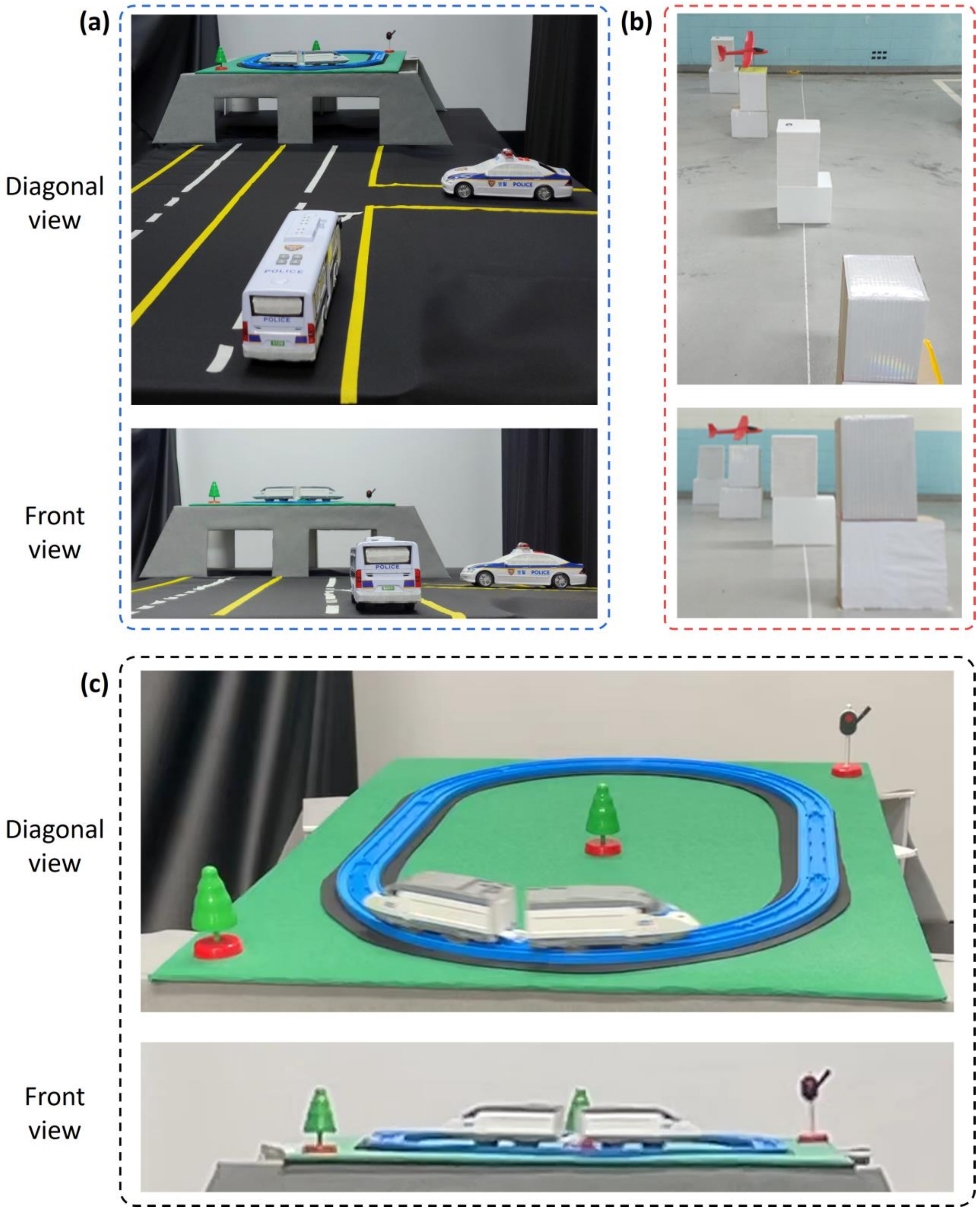

Supplementary Fig. 17. Photographs of target configurations for 4D coherent ranging of (a) Scene A, **(b)** Scene B, and **(c)** Scene C.

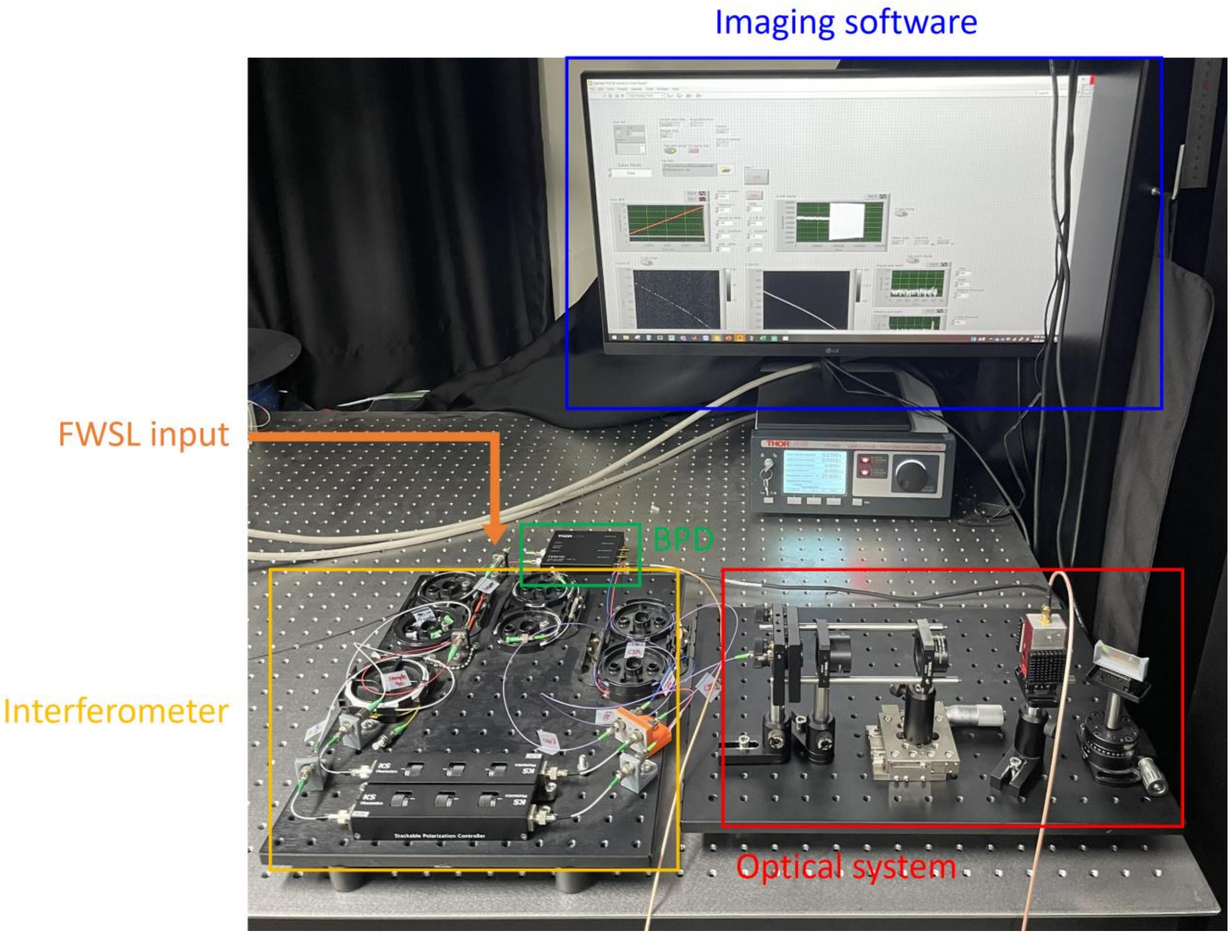

Supplementary Fig. 18. Photograph of the experimental setup.

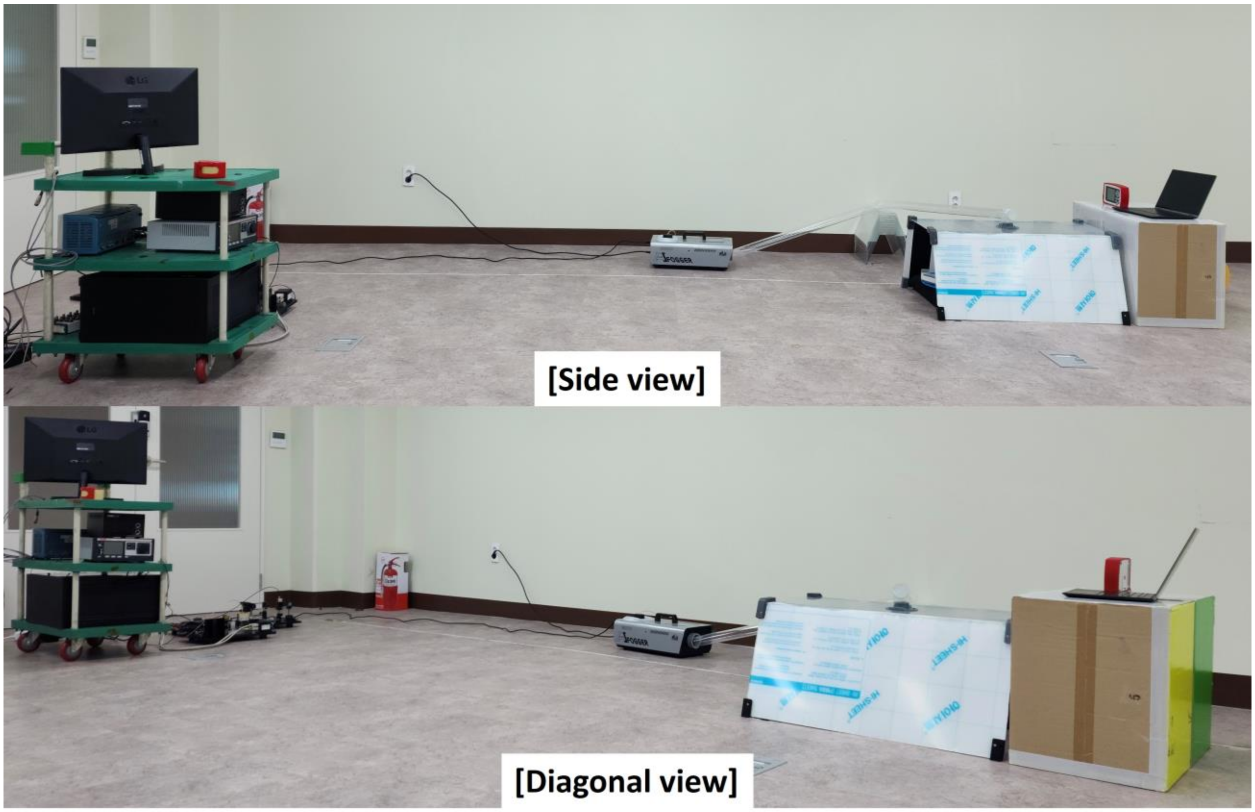

Supplementary Fig. 19. Photograph of the actual measurement scene during the real-time 4D coherent ranging in a simulated fog environment.

References

1. Okano, M. & Chong, C. Swept Source Lidar: simultaneous FMCW ranging and nonmechanical beam steering with a wideband swept source. *Opt Express* **28**, 23898 (2020).

2. Qian, R. *et al.* Video-rate high-precision time-frequency multiplexed 3D coherent ranging. *Nat Commun* **13**, (2022).

3. Haiping, G., Chenhao, W., Fei, W., Lijing, Z. & Chengwen, X. Study on the dynamic mode stability of grating-feedback external cavity diode lasers. *Laser Phys* **26**, 045002 (2016).

4. Gong, H., Liu, Z., Zhou, Y. & Zhang, W. Extending the mode-hop-free tuning range of an external-cavity diode laser by synchronous tuning with mode matching. *Appl Opt* **53**, 7878 (2014).

5. Levin, L. Mode-hop-free electro-optically tuned diode laser. *Opt Lett* **27**, 237 (2002).

6. Bogatov, A., Eliseev, P. & Sverdlov, B. Anomalous interaction of spectral modes in a semiconductor laser. *IEEE J Quantum Electron* **11**, 510–515 (1975).

7. Ogasawara, N. & Ito, R. Longitudinal Mode Competition and Asymmetric Gain Saturation in Semiconductor Injection Lasers. II. Theory. *Jpn J Appl Phys* **27**, 615 (1988).

8. Yamada, M. Theoretical analysis of nonlinear optical phenomena taking into account the beating vibration of the electron density in semiconductor lasers. *J Appl Phys* **66**, 81–89 (1989).

9. F. N. Timofeev M. S. Shatalov S. A. Gurevich P. Bayvel R. Wyatt I. Lealman R. Kashyap, G. S. S. Experimental and Theoretical Study of High Temperature-Stability and Low-Chirp 1.55 μm Semiconductor Laser with an External Fiber Grating. *Fiber and Integrated Optics* **19**, 327–353 (2000).

10. Trutna, W. R. & Stokes, L. F. Continuously tuned external cavity semiconductor laser. *Journal of Lightwave Technology* **11**, 1279–1286 (1993).

11. Chong, C., Suzuki, T., Morosawa, A. & Sakai, T. Spectral narrowing effect by quasi-phase continuous tuning in high-speed wavelength-swept light source. *Opt Express* **16**, 21105 (2008).

12. Okoshi, T., Kikuchi, K. & Nakayama, A. Novel method for high resolution measurement of laser output spectrum. *Electron Lett* **16**, 630 (1980).

13. Mercer, L. B. 1/f frequency noise effects on self-heterodyne linewidth measurements. *Journal of Lightwave Technology* **9**, 485–493 (1991).

14. Jang, H. *et al*. Simultaneous distance and vibration mapping of FMCW-LiDAR with akinetic external cavity diode laser. *Opt Lasers Eng* **160**, 107283 (2023).

15. Park, S., Rim, S., Kim, Y. & Lee, B. H. Noncontact photoacoustic imaging based on optical quadrature detection with a multiport interferometer. *Opt Lett* **44**, 2590 (2019).

16. Choma, M. A., Yang, C. & Izatt, J. A. Instantaneous quadrature low-coherence interferometry with 3×3 fiber-optic couplers. *Opt Lett* **28**, 2162 (2003).

17. Suyama, S., Ito, H., Kurahashi, R., Abe, H. & Baba, T. Doppler velocimeter and vibrometer FMCW LiDAR with Si photonic crystal beam scanner. *Opt Express* **29**, 30727 (2021).
