# Supplementary material for: Spatio-spectral 4D coherent ranging using a flutter-wavelength-swept laser
Source: Nat Commun. 2024 Feb 6;15:1110. doi: 10.1038/s41467-024-45297-w (PMC10847489; doi:10.1038/s41467-024-45297-w)
Supplement: Supplementary file 3 — Description of Additional Supplementary Files [file 41467_2024_45297_MOESM3_ESM.docx]

**Description of Additional Supplementary Files**

**File Name: Supplementary Video 1
Description:** Real-time 4D distance and velocity video of Scene C.

**File Name: Supplementary Video 2
Description:** Real-time 4D distance and velocity video of Scene D in a challenging environment.
